# Mechanochemical Synthesis of Cross-Linked Chitosan and Its Application as Adsorbent for Removal of Per- and Polyfluoroalkyl Substances from Simulated Electroplating Wastewater

**DOI:** 10.3390/ma17123006

**Published:** 2024-06-19

**Authors:** Giovanni Cagnetta, Zhou Yin, Wen Qiu, Mohammadtaghi Vakili

**Affiliations:** 1 Faculty of Geosciences and Environmental Engineering, Southwest Jiaotong University, Chengdu 610031, China; 2 State Key Joint Laboratory of Environment Simulation and Pollution Control (SKLESPC), Beijing Key Laboratory for Emerging Organic Contaminants Control, School of Environment, Tsinghua University, Beijing 100084, China; yinzhou1029@126.com (Z.Y.); qqqiuwen@163.com (W.Q.); 3 ORLEN UniCRE, a.s., Revoluční 1521/84, 400 01 Ústí nad Labem, Czech Republic

**Keywords:** chitosan, mechanochemical synthesis, cross-linking, adsorption, PFAS

## Abstract

Chitosan is a promising adsorbent for removing a wide range of pollutants from wastewater. However, its practical application is hindered by instability in acidic environments, which significantly impairs its adsorption capacity and limits its utilization in water purification. While cross-linking can enhance the acid stability of chitosan, current solvent-based methods are often costly and environmentally unfriendly. In this study, a solvent-free mechanochemical process was developed using high-energy ball milling to cross-link chitosan with various polyanionic linkers, including dextran sulfate (DS), poly[4-styrenesulfonic acid-co-maleic acid] (PSSM), and tripolyphosphate (TPP). The mechanochemically cross-linked (MCCL) chitosan products exhibited superior adsorption capacity and stability in acidic solutions compared to pristine chitosan. Chitosan cross-linked with DS (Cht-DS) showed the highest Reactive Red 2 (RR2) adsorption capacity, reaching 1559 mg·g^−1^ at pH 3, followed by Cht-PSSM (1352 mg·g^−1^) and Cht-TPP (1074 mg·g^−1^). The stability of MCCL chitosan was visually confirmed by the negligible mass loss of Cht-DS and Cht-PSSM tablets in pH 3 solution, unlike the complete dissolution of the pristine chitosan tablet. The MCCL significantly increased the microhardness of chitosan, with the order Cht-DS > Cht-PSSM > Cht-TPP, consistent with the RR2 adsorption capacity. When tested on simulated rinsing wastewater from chromium electroplating, Cht-DS effectively removed Cr(VI) (98.75% removal) and three per- and polyfluoroalkyl substances (87.40–95.87% removal), following pseudo-second-order adsorption kinetics. This study demonstrates the potential of the cost-effective and scalable MCCL approach to produce chitosan-based adsorbents with enhanced stability, mechanical strength, and adsorption performance for treating highly acidic industrial wastewater containing a mixture of toxic pollutants.

## 1. Introduction

Chitosan, obtained from the deacetylation of chitin found in shrimp and other crustacean shells using strong alkali solutions, is a polymer composed of randomly N-acetylated units of glucosamine (poly[β-(1→4)-D-glucosamine]). This versatile polysaccharide finds extensive applications across various industries [1]. In agriculture, chitosan functions as a biodegradable film that enhances plant growth, improves soil quality, and offers crop protection against pests and diseases [2]. In the food industry, it acts as a preservative due to its antimicrobial properties and is used as a dietary supplement for weight loss and cholesterol management [3]. Within the cosmetics sector, chitosan is incorporated into skincare products for its moisturizing and anti-inflammatory attributes [4]. It is employed in wound dressings and bandages in the medical field to facilitate wound healing, reduce bleeding, and prevent infection [5]. Nevertheless, its most notable impact has been in water treatment, where it binds various pollutants and forms flocs, contributing significantly to the growth of the chitosan market in recent years [6].

Chitosan’s high surface area, cationic charge, and amino groups facilitate this biopolymer’s efficient adsorption of various pollutants, including heavy metals, dyes, PFASs, and other organic compounds, through mechanisms such as ion exchange and complexation [7,8]. Its intrinsic versatility also allows modifications to enhance its adsorption capacity and selectivity. A range of physical, biological, and chemical methods have been developed to improve or introduce new properties to chitosan [9]. In the context of water treatment, chemical modifications such as grafting, impregnation, oxidation, and cross-linking are frequently employed to enhance the adsorption properties of chitosan due to its reactivity [10]. For instance, oxidation using mild oxidants has been extensively investigated due to its simplicity, resulting in the introduction of oxygen-containing functional groups and the reduction of the average molecular weight of chitosan. These modifications increase the number of adsorption sites and reduce the crystallinity of the polymer, thereby providing access to adsorption sites within the inner layers of the particle. Empirical studies indicate that oxidized chitosan exhibits enhanced adsorption capacity for various pollutants, attributed to its increased hydrophilicity and enhanced electrostatic interactions with the adsorbate [1].

Similarly, the process of cross-linking chitosan entails the formation of bridging bonds among its chains, leading to a network structure that significantly enhances its mechanical strength and stability [6]. Agents such as glutaraldehyde and epichlorohydrin act as cross-linking agents, augmenting chitosan’s pollutant adsorption capacity by increasing its surface area and porosity [11,12]. These modifications also improve chitosan’s resistance to degradation, rendering it more durable in challenging environments, such as low-pH aqueous solutions. However, due to their reliance on solvent-based methodologies, these chemical techniques are not easily scalable for producing large quantities of chitosan-based adsorbents. The extensive use of solvents, typically water, in these processes results in high energy costs for drying the final product, as well as the need for regeneration and proper disposal of the solvents used as cross-linking reaction media and for washing the final product, which become contaminated by residual reagents and by-products. These operations clearly have substantial environmental and economic impacts. Consequently, a solvent-free approach is essential to render these chemical methods more cost-effective for producing more efficient adsorbents.

Mechanochemical methodologies, particularly high-energy ball milling, are now widely recognized as viable options for synthesizing materials through solid-state approaches [13,14]. These methodologies offer numerous benefits, including high efficiency, simple process design, and a wide range of machinery typologies that are often available on the market at moderate prices [15,16]. High-energy ball milling is a versatile technique that can reduce particle size, increase specific surface area, and lead to significant physicochemical modifications in the milled materials [17]. During this process, the intense impacts from milling tools can break chemical bonds, reduce crystallinity, induce or enhance amorphization, and increase the density of surface functional groups in solid materials [18]. This can subsequently improve the adsorption behavior of adsorbents, including chitosan.

Previous findings from our research group indicate that the sole high-energy ball milling treatment can augment the specific surface area of chitosan while concurrently reducing its average molecular weight via chain scission [19]. Moreover, when this biopolymer undergoes mechanochemical treatment in the presence of a limited quantity of a finely powdered mild oxidizing agent, the extensive generation of oxygenated groups and consequential chain scission notably amplify the adsorption characteristics of the oxidized chitosan compared to the pristine polysaccharide [20]. These findings strongly imply that additional reactions of chitosan can be facilitated through a solid-state approach. One particularly compelling yet unexplored reaction pathway of this biopolymer is cross-linking, which holds significant promise across various domains. Specifically, in wastewater treatment, reinforcing the mechanical integrity of chitosan becomes imperative to fabricate efficient adsorption beds for industrial adsorption columns. Furthermore, resilience against highly acidic pH environments, prevalent in industrial effluents, emerges as a critical attribute for utilizing modified chitosan as a cost-effective adsorbent.

To address these requirements, a mechanochemical method was developed in this investigation to cross-link chitosan via a straightforward one-step synthesis. The aims of this study are: (1) to ascertain the viability of chitosan cross-linkage through direct interaction with three varieties of cross-linkers (tripolyphosphate, poly[4-styrenesulfonic acid-co-maleic acid], and dextran sulfate) in the solid-state employing a planetary ball mill; (2) to evaluate its endurance in a highly acidic environment (pH = 3) in both powdered and pelletized forms; and (3) to appraise the adsorption efficacy of the most optimal cross-linked chitosan with simulated chrome-electroplating wastewater from rinsing operations.

The electroplating industry is a prominent contributor to environmental degradation due to its substantial discharge of heavy metals, notably chromium, and per- and poly-fluoroalkyl substances (PFAS). Hexavalent chromium, acknowledged for its profound toxicity and carcinogenicity [21], poses a significant threat to human health, particularly among electroplating industry personnel. The burgeoning evidence implicating PFAS in adverse health outcomes underscores the urgency of addressing these pollutants, given their ubiquitous presence in aquatic environments globally [22,23,24]. This study endeavors to confront these pressing environmental concerns by developing a more productive and sustainable wastewater treatment strategy. The crux of the challenge lies in effectively managing the disposal of spent chrome-plating baths and rinsing water utilized to clean plated artifacts, both of which harbor elevated concentrations of Cr(VI) resistant to conventional treatment methods. Conventional approaches entail acidifying bath water with sulfur compounds to effectuate the reduction of hexavalent chromium to its elemental state, followed by separation through sedimentation. The treatment of rinsing wastewater proves even more intricate due to the co-occurrence of diverse heavy metals (e.g., Cr(III), Ni(II), Cu(II), etc.) and PFAS, utilized as chrome mist suppressants, alongside its acidic milieu and heightened sulfate content [25,26]. Present treatment protocols primarily focus on Cr(VI) reduction to Cr(III) and subsequent pH adjustment to induce metal hydroxide precipitation, although they fall short in addressing PFAS removal. Consequently, the imperative for a novel, streamlined technology capable of concurrently removing Cr(VI) and PFAS while optimizing resource utilization and time efficiency becomes evident. To this end, our study proposes the utilization of mechanochemically cross-linked chitosan as a promising adsorbent for the purpose mentioned earlier.

## 2. Materials and Methods

### 2.1. Materials

**Figure A1:**
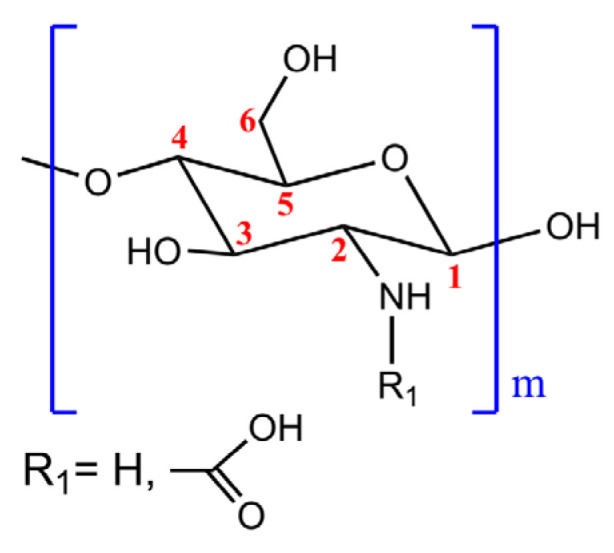
Chitosan.

**Figure A2:**
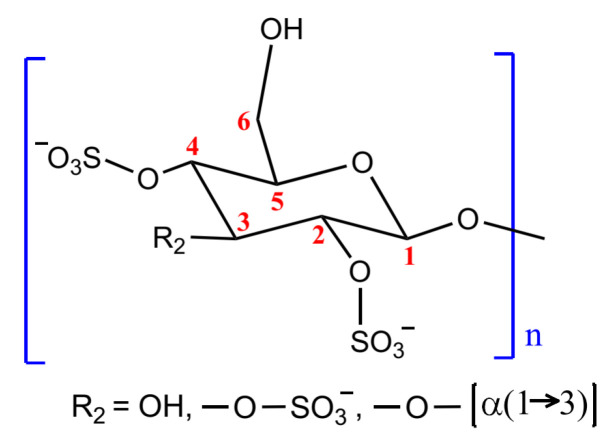
Dextran sulfate.

**Figure A4:**
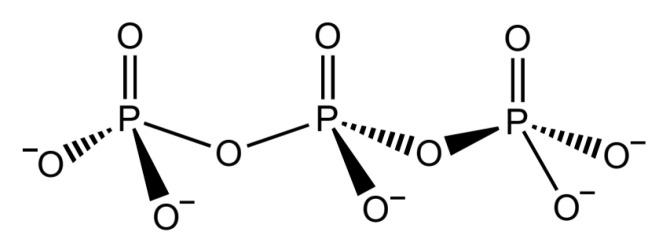
Tripolyphosphate.

**Figure A5:**
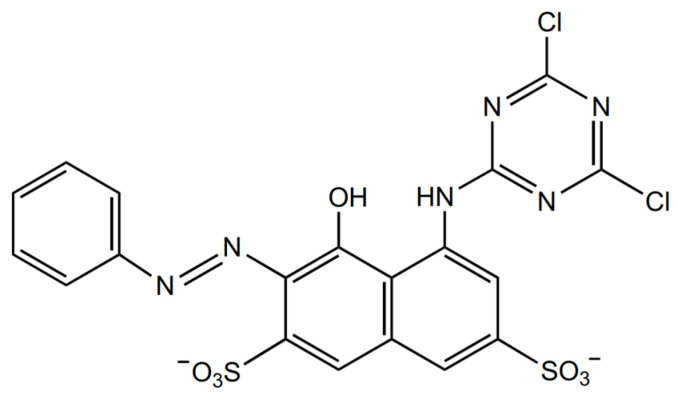
Reactive red 2.

**Figure A6:**
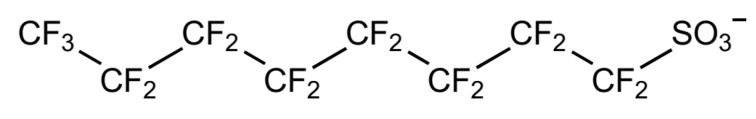
Perfluorooctane sulfonate.

**Figure A7:**
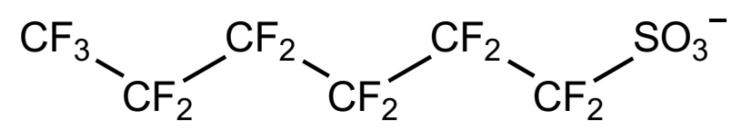
Perfluorohexane sulfonate.

**Figure A8:**
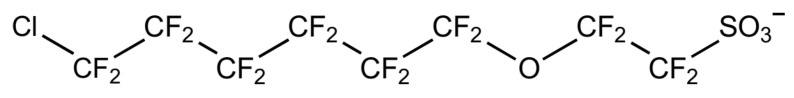
F-53B.

Medium molecular weight chitosan (75–85% deacetylated; generic chemical structure is shown in Figure A1, Appendix A) was purchased from Sigma-Aldrich. Dextrane sulfate sodium salt (DS; Figure A2), poly[4-styrenesulfonic acid-co-maleic acid] sodium salt (PSSM, SS:MA = 3:1; Figure A3), and tripolyphosphate sodium salt (TPP; Figure A4) were obtained from Sigma Aldrich (Beijing, China) and utilized as cross-linkers. Reactive Red 2 (RR2, molecular mass: 615.33 g·mol^−1^; Figure A5) was obtained from Rhawn Chemical Reagents (Shanghai, China) and was used to test the adsorption of cross-linked chitosan products for the optimization of high-energy milling parameters. Perfluorooctane sulfonate potassium salt (PFOS, molecular mass of the anion: 538.22 g·mol^−1^, >98%, Sigma-Aldrich Co., Burlington, MA, USA; Figure A6), perfluorohexane sulfonate potassium salt (PFHxS, molecular mass of the anion: 399.11 g·mol^−1^, >98%, Wuhan Silworld Chemical Ltd., Wuhan, China; Figure A7), and 2-[(6-chloro-dodecafluorohexyl)oxyl]-perfluoroethanesulfonate potassium salt (a Chinese alternative to PFOS with commercial name F-53B, mainly employed as mist suppressant in chrome plating industry, molecular mass of the anion: 531.58 g·mol^−1^, >98%, Shanghai Synica Corp, Shanghai, China; Figure A8), potassium dichromate (K_2_Cr_2_O_7_, molecular mass of the anion: 215.99 g·mol^−1^, >98%, Sigma-Aldrich Co., Burlington, MA, USA), and chromium trichloride (CrCl_3_, molecular mass of the cation: 52.00 g·mol^−1^, >98%, Sigma-Aldrich Co., Burlington, MA, USA) were used to prepare the simulated wastewater derived from rinsing operations in the chrome-electroplating industry. Deionized water was produced by a Milli-Q Integral water purification system (Millipore, Bedford, MA, USA).

### 2.2. Mechanochemical Cross-Linking Experiments

Mechanochemical cross-linking (MCCL) was conducted using a planetary ball mill (Model QM-3SP2, Nanjing University Instrument Corporation, Nanjing, China) as the mechanochemical reactor. Chitosan and a cross-linker were combined in powder form, with varying mass percentages (10%, 20%, 30%, 40%, and 50%) of the latter, constituting a total weight of 2 g. These mixtures were introduced into stainless steel milling jars (70 cm^3^) along with 128 g of stainless-steel balls (∅5 mm). The high-energy mill’s central disc rotated at a speed of 225 rpm, maintaining a central disc-to-jar rotation ratio of −2. Following specific milling durations (2, 4, and 6 h), the samples were retrieved and stored in a desiccator before undergoing characterization and adsorption tests.

#### Characterization of the Milled Products

Fourier-transform infrared spectroscopy with attenuated total reflectance (FTIR-ATR; FTS3000, Digilab, MA, USA) spanning the range 4000–400 cm^−1^ was conducted to discern alterations in functional groups within the products. Scanning electron microscopy (SEM, LEO-1530, LEO, Zeiss, Oberkochen, Germany) was employed to visualize the morphology of the milled products. The specific surface area of the milled samples was determined utilizing the Brunauer-Emmett-Teller (BET) method using a gas adsorption instrument (Autosorb iQ, Quantachrome Corp., Boynton Beach, FL, USA). Then, ^13^C cross-polarization under magic-angle spinning nuclear magnetic resonance (CP-MAS NMR) was carried out using a JNM-ECZ600R console (JEOL Ltd., Tokyo, Japan) with a 150 MHz Larmor frequency. A double resonance probe supporting zirconia MAS rotors (3.2 mm outer diameter) was utilized at a MAS spinning frequency of 12 kHz, 2 ms contact time, and 3 s relaxation delay. The microhardness of pristine chitosan and milled samples in the form of circular tablets (10 mm diameter, 1 g cross-linked chitosan, prepared with a manual press at 10 MPa for 10 min), was evaluated using an automated micro-Vickers hardness tester (Shimadzu, HMV-2, Tokyo, Japan).

### 2.3. Adsorption Experiments

#### 2.3.1. Assessment of the Adsorption Properties of Mechanochemically Cross-Linked Chitosan Products

A series of batch tests were conducted to determine the adsorption properties of the MCCL products. Without further purification, milled products (5 mg) were placed in 100 mL Erlenmeyer flasks containing 50 mL of RR2 aqueous solution (100 mg·L^−1^). The initial pH was adjusted to 3 using 1 M HCl aqueous solution, and the flasks were agitated on a rotary shaker at 160 rpm and at room temperature (22 °C) for 24 h. After filtration of approximately 1 mL of the solution sample through 0.22 μm nylon syringe filters, the residual RR2 concentration was quantified using a spectrophotometric method (employing a DR 5000, Hach Lange, Düsseldorf, Germany), measuring the absorption at a wavelength of λ = 538 nm. In another set of batch experiments to evaluate the influence of initial pH on the adsorption capacity of the milled products, the pH was adjusted by dropwise addition of 1 M HCl or 1 M NaOH while maintaining the aforementioned methodology unchanged.

A reusability test was conducted by placing 1 tablet of MCCL products in 100 mL Erlenmeyer flasks containing 50 mL of RR2 aqueous solution (100 mg·L^−1^) with the pH adjusted to 3. After 24 h of agitation at 160 rpm and room temperature (22 °C), a solution sample was withdrawn and filtered through 0.22 μm nylon syringe filters, and RR2 was quantified using the aforementioned spectrophotometric method. The tablet was then carefully removed and placed in a new 100 mL Erlenmeyer flask containing 50 mL of 0.1 M NaOH regenerating solution. After washing for 4 h, the regenerated tablet was transferred to an Erlenmeyer flask with fresh RR2 solution for the subsequent adsorption cycle. This adsorption–regeneration cycle was repeated five times.

#### 2.3.2. Adsorption Treatment of Simulated Chrome-Plating Wastewater

Based on the concentrations of Cr(VI), Cr(III), and PFAS as documented in the studies of Liu et al. [25] and Qu et al. [26], the synthetic chrome-electroplating wastewater was meticulously formulated in accordance with the reported quantities delineated in Table 1.

The simulated wastewater was treated by adding a selected MCCL chitosan product (5 mg) to 50 mL of wastewater in a 100 mL Erlenmeyer flask. The flask was agitated on a rotary shaker at 160 rpm and at room temperature (22 °C). Approximately 1 mL of the sample was withdrawn and filtered at defined time intervals using a 0.22 μm polyether sulfone syringe filter. Subsequently, it was refrigerated at 4 °C and subjected to quantification analyses.

The total chromium concentration was determined using an atomic absorption spectrometer (AA240FS, Varian, Palo Alto, CA, USA). Hexavalent chromium was analyzed via the spectrophotometric method (using a DR 5000, Hach Lange, Germany), with absorption measured at λ = 350 nm [27]. PFOS, PFHxS, and F-53B were quantified using an LC/MS system (6475 Triple Quadrupole LC/MS System, Agilent, Palo Alto, CA, USA), employing an Agilent ZORBAX Eclipse Plus RRHD C18 column (2.1 × 50 mm, 1.8 μm) with a guard column (Agilent ZORBAX Eclipse Plus C18, 2.1 × 5 mm, 1.8 μm). The column oven temperature was maintained at 45 °C, and the mobile phase comprised ammonium acetate (5 mM) and methanol (70%*v*/*v*) injected at a feeding rate of 0.4 mL min^−1^. MS/MS settings were optimized before obtaining calibration curves for each perfluoroalkyl chemical. Sulfate quantification was performed via ion chromatography (ICS-2000, Dionex, Sunnyvale, CA, USA).

## 3. Results and Discussion

### 3.1. Mechanochemical Cross-Linking of Chitosan

In the investigation of solid-state cross-linking of chitosan, three polyanionic linkers (TPP, PSSM, and DS) were co-milled with the polysaccharide in a planetary ball mill. These linkers can establish multiple ionic bonds with the protonated amino groups of chitosan, thereby forming a bond network within the milled product. The pivotal parameters scrutinized to monitor the advancement of the reaction encompassed milling duration and the quantity of cross-linker (expressed as a percentage of the total mass in the reaction mixture). These parameters exert a substantial influence on the kinetics of the mechanochemical reaction. Milling duration plays a crucial role in determining the extent of mechanical energy introduced into the reaction mixture, whereas the quantity of reagent is linked to the reaction rate through a rate equation [28]. Generally, a prolonged milling duration and a higher quantity of reagent lead to enhanced conversion of the mechanochemical reaction. This correlation has been substantiated through numerous reactions involving both organic and inorganic compounds conducted in high-energy mills [29,30].

The success of MCCL reaction was assessed through an adsorption examination of the milled products conducted in an RR2 solution with an initial pH of 3; the concentration of unadsorbed RR2 in the solution was measured after 24 h. In an acidic environment, the chitosan glycosidic bond undergoes hydrolysis, significantly compromising the adsorption capacity of the biopolymer. Therefore, the more effective the cross-linking, the more resilient the structure of the product at low pH, and, consequently, the greater the RR2 adsorption capacity of the specimen.

#### 3.1.1. Effect of the Cross-Linker Type and Amount

**Figure 1:**
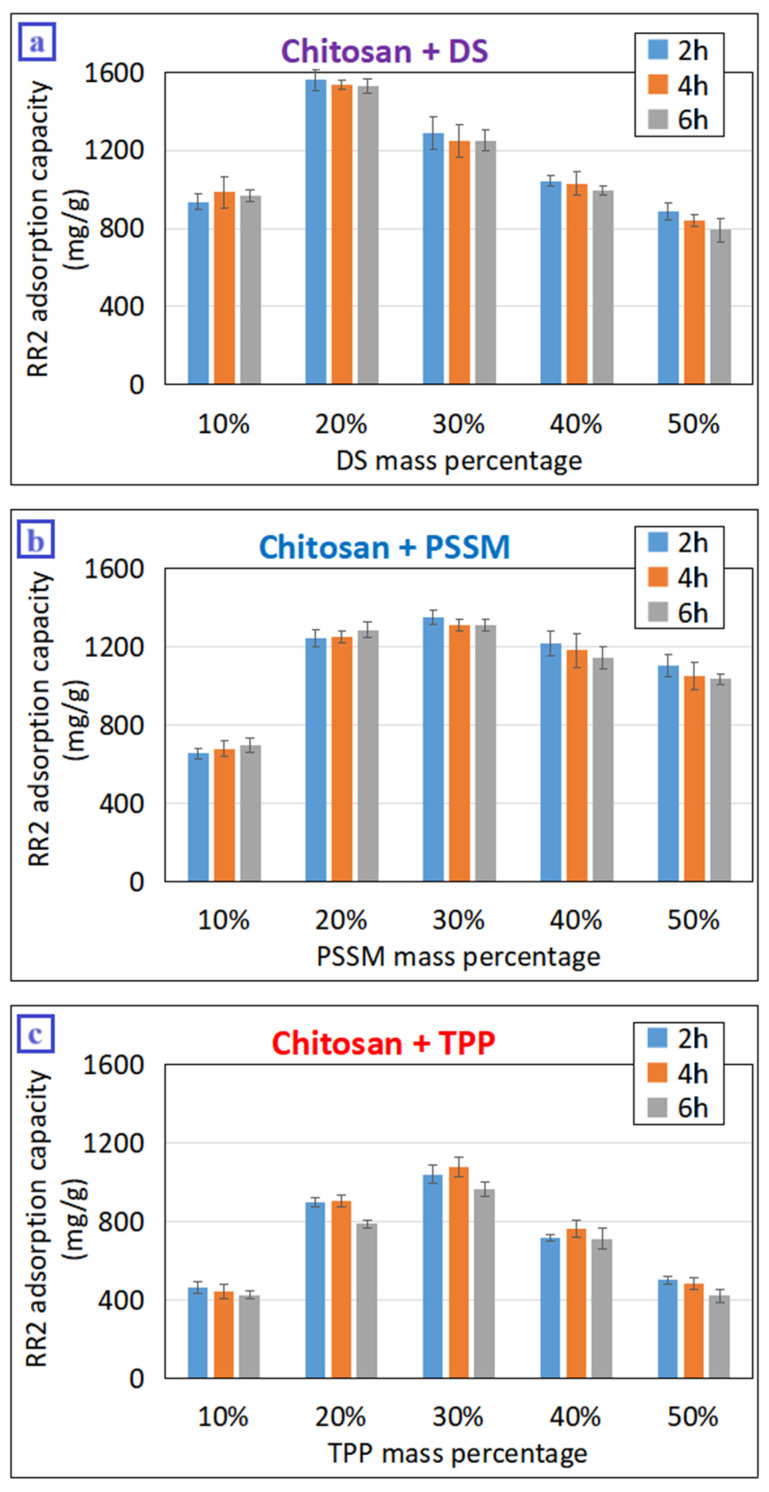
The RR2 adsorption capacity of mechanochemically cross-linked chitosan adsorbents synthesized with (**a**) DS, (**b**) PSSM, and (**c**) TPP as cross-linkers, varying milling time and linker weight percentage. Adsorption test conditions: RR2 = 50 mg·L^−1^, 5 mg oxidized product in 100 mL solution, initial pH = 3, contact time = 24 h.

Under optimized conditions of the mechanochemical reaction, chitosan milled with 20%*w*/*w* DS for 2 h (named Cht-DS) exhibited the highest adsorption capacity for RR2, amounting to 1559 mg·g^−1^ (Figure 1a). The adsorbent synthesized via 2-h high-energy milling with 30%*w*/*w* PSSM (Cht-PSSM) demonstrated a slightly lower RR2 uptake at 1352 mg·g^−1^, while the product from 4 h milling with 30%*w*/*w* TPP (Cht-TPP) showed a maximum adsorption capacity of 1074 mg·g^−1^. These experimental findings validate the efficacy of the proposed MCCL method in stabilizing chitosan in highly acidic solutions, resulting in significant RR2 adsorption capacities irrespective of the chosen cross-linker.

The most efficient adsorbent was synthesized with DS, likely owing to its relatively high number of sulfonated groups per monomer, thereby enhancing its adsorption capacity. In contrast, PSSM, being a copolymer of 4-styrenesulfonic acid and maleic acid, contains a lower degree of sulfonation, potentially compromising structural stability and, consequently, reducing adsorption capacity [31]. This necessitates a higher proportion of PSSM to attain optimal adsorption capacity, presumably due to the need for greater structural stability (i.e., a larger number of sulfonate groups). Regarding TPP, the lower acidity of –PO_3_H_2_, compared to –SO_3_H in DS, likely results in weaker ionic bonding and inferior resistance in acidic solutions, thereby leading to its lower RR2 adsorption capacity [32]. This observation elucidates the relatively high TPP mass percentage (30%*w*/*w*) required to achieve optimum adsorption capacity.

When analyzing the adsorption capacity variation with the mass percentage of each cross-linker, an optimal value that maximizes RR2 adsorption capacity is consistently discernible (Figure 1). Clearly, percentages below this threshold were insufficient to achieve complete cross-linking of the corresponding amount of chitosan, resulting in inferior structural stability. Consequently, unlinked chitosan was readily hydrolyzed at a pH of 3 during the adsorption test, leading to diminished adsorption performance. Conversely, exceeding the optimum cross-linker percentage led to a decline in adsorption capacity, attributable to an excess of unreacted linkers that reduce the fraction of effective adsorbent, particularly the number of amine groups [33].

#### 3.1.2. Effect of the High-Energy Milling Time

Under the tested milling conditions with fixed operating parameters (i.e., rotation speed, ball-to-powder charge ratio, jar filling ratio, etc.), milling time was chosen as the sole factor capable of controlling the amount of mechanical energy imparted to the reaction mixture and, consequently, influencing the progress of the reaction. Analogous to the cross-linker percentage, the optimal milling time facilitated the attainment of the highest adsorption capacity of the product (Figure 1). Specifically, Cht-DS and Cht-PMMS required 2 h to maximize RR2 uptake on chitosan, while Cht-TPP necessitated 4 h. The prolonged milling time required for the mechanochemical reaction with TPP, coupled with its higher mass percentage in the mixture, likely stems from the lower reactivity of phosphate compared to sulfonate under high-energy milling conditions. In essence, the phosphate groups of TPP required an extended reaction time to establish a sufficient number of bonds with chitosan’s NH_3_^+^ to ensure structural stability and maximize adsorption capacity.

Generally, an optimal milling time maximizes the RR2 adsorption capacity, as shown in Figure 1, for 4 h of milling with 10% DS and 30% TPP. This optimal time arises from various opposing processes occurring during milling that can either enhance or diminish the final adsorption capacity of the product. The principal process is the MCCL reaction, which involves forming or rupturing ionic bonds due to the input of sufficient or excessive energy into the system. Furthermore, particle comminution and agglomeration are fundamental opposing processes that significantly influence the specific surface area of chitosan, thereby determining its adsorption capacity [19]. Conversely, amorphization of chitosan increases unabatedly and profoundly affects numerous properties of the polysaccharide [34], including enhancing its adsorption ability [19]. Overall, the optimal milling time corresponds to the amount of mechanical energy that maximizes the RR2 adsorption capacity as a result of all these aforementioned processes.

### 3.2. Characterization of Mechanochemically Cross-Linked Chitosan Products

#### 3.2.1. Surface Properties

**Figure 2:**
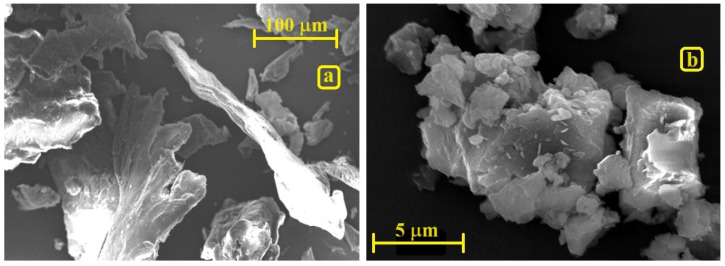
SEM images of (**a**) pristine chitosan and (**b**) Cht-DS.

SEM images demonstrate that chitosan particles, initially varying in size from 0.1 to 1 mm (Figure 2a), are reduced to approximately 10 μm irrespective of the cross-linker type employed (Figure 2b depicts only Cht-DS, as analogous morphology is observed in other cross-linked samples). The milled particles manifest as aggregates or agglomerates of smaller particles, suggesting that while chitosan particles diminish in size during milling, they concurrently undergo a process of aggregation and agglomeration, culminating in the formation of larger particles. Electrostatic interactions (aggregates) or chemical bonds (agglomerates) bind these minute particles [35].

The specific surface area of chitosan, as determined by the BET method, more than doubles due to exclusive milling, increasing from 0.922 m^2^ g^−1^ for pristine chitosan to 2.073 m^2^ g^−1^ for chitosan milled for 2 h (Table 2). This observation is consistent with the formation of aggregates or agglomerates exhibiting an irregular surface morphology. While all MCCL chitosan samples manifest a diminished specific surface area compared to chitosan milled for 2 h, this phenomenon can be ascribed to the compact structure and a higher percentage of aggregates in the cross-linked specimens, alongside a probable “dilution effect” induced by the cross-linker. The diminished value observed in the TPP cross-linked sample may also be attributed to an extended milling duration, culminating in material structural collapse and a consequent reduction in surface area [19].

#### 3.2.2. Fourier Transform Infrared Spectroscopy Analysis

**Figure 3:**
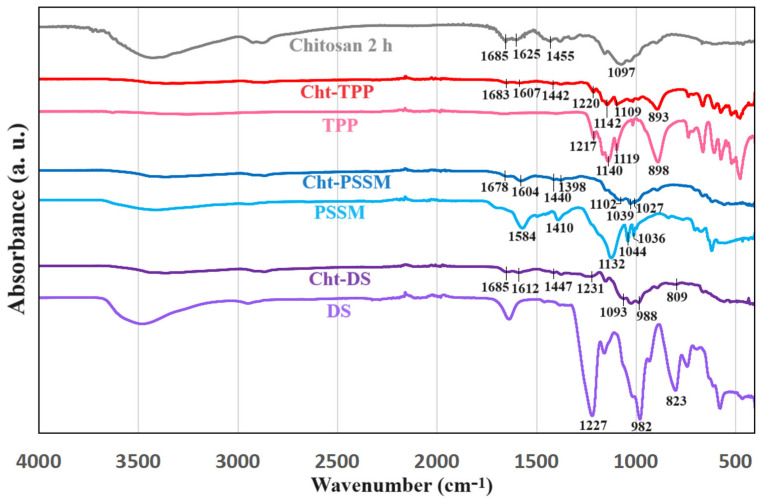
FTIR-ATR spectra of chitosan milled for 2 h, the investigated cross-linkers (DS, PSSM, and TPP), and the corresponding MCCL reaction products (Cht-DS, Cht-PSSM, and Cht-TPP).

During high-energy ball milling treatment, chitosan typically undergoes minimal chemical modifications [36]. Consequently, the FTIR spectrum of the milled sample, when juxtaposed with that of the original polysaccharide, exhibits no discernible shifts in the peaks. Instead, a generalized broadening and decrease in intensity occur due to amorphization, characterized by the random distortion of chemical bonds [1]. Illustrated in Figure 3 are the spectra of chitosan milled for 2 h, alongside those of the investigated cross-linkers and their resultant products. While a comprehensive list of chitosan’s unique signals was provided by Vakili et al. [6], here it suffices to note that the prominent band at 1097 cm^−1^ corresponds to C-O vibrations of the pyranosic ring, the doublet at 1625 and 1685 cm^−1^ corresponds to N-H and primary amide vibrations, respectively, and the peak at 1455 cm^−1^ is attributed to NH_2_ stretching.

In the fingerprint region, the spectrum of dextran sulfate (DS) exhibits three bands associated with symmetric vibrations of C–O–SO_3_^−^ groups (823 cm^−1^), C–O vibrations of the pyranosic ring, and glycosidic bonds (982 cm^−1^), and asymmetric stretching of S=O (1227 cm^−1^) [37]. Conversely, the Cht-DS spectrum displays a slight red shift in the peaks associated with chitosan’s amine and DS’ sulfonate groups. Specifically, the wave numbers of the N–H (1625 cm^−1^) and NH_2_ (1455 cm^−1^) signals decrease to 1612 and 1447 cm^−1^, respectively, while that of C–O–SO_3_^−^ shifts from 823 to 809 cm^−1^. This shift indicates a stiffening of these bonds, likely due to –NH_3_^+^∙∙∙^−^O_3_S– ionic bonding or –^+^NH–H∙∙∙^−^O–SO_2_– hydrogen bonding [38].

In the fingerprint region, PSSM exhibits characteristic peaks related to the sulfonate group, including a prominent band at 1132 cm^−1^ and a doublet at 1044 and 1036 cm^−1^, attributed to the symmetric and asymmetric stretching of –SO_3_^−^ (1227 cm^−1^) [37]. Additionally, signals at 1584 and 1410 cm^−1^ are identified as C=C stretching vibrations of the benzenic ring. However, the Cht-PSSM spectrum presents challenges in interpretation as the distinctive peaks overlap with those of chitosan. It is cautiously assumed that the doublet observed at 1039 and 1027 cm^−1^ corresponds to –SO_3_^−^ asymmetric vibrations, indicating potentially reduced vibration energy due to ionic bonding with chitosan’s ammonium groups. Similarly, the minor peak at 1440 cm^−1^ may correspond to –NH_2_ stretching shifted from 1455 cm^−1^ rather than the C=C signal shifted from 1410 cm^−1^, suggesting a potential strengthening of the amine bond due to ionic or hydrogen bonding interactions, likely involving sulfonates and carboxylates of the PSSM [39].

The distinctive features of TPP in the fingerprint region encompass a peak at 898 cm^−1^ corresponding to P–O–P bridge stretching, and a broad band with three signals at 1119, 1140, and 1217 cm^−1^ attributed to the vibration of –PO_3_^2−^, O–P=O, and P=O, respectively [40]. Cht-TPP was synthesized subsequent to a prolonged milling duration of 4 h and a heightened proportion of the cross-linker (30%*w*/*w*), culminating in a reduction in chitosan signal and accentuation of the TPP peaks. The vibration signals of –PO_3_^2−^ and P–O–P in the Cht-TPP spectrum underwent a redshift to 1109 and 893 cm^−1^, correspondingly, while the peaks assigned to N–H and –NH_2_ shifted to 1607 and 1442 cm^−1^, respectively. These shifts imply the occurrence of ion/hydrogen bonding between the phosphate anions of TPP and the ammonium cations of chitosan, indicative of the manifestation of the MCCL product.

#### 3.2.3. ^13^C Nuclear Magnetic Resonance Analysis

**Figure 4:**
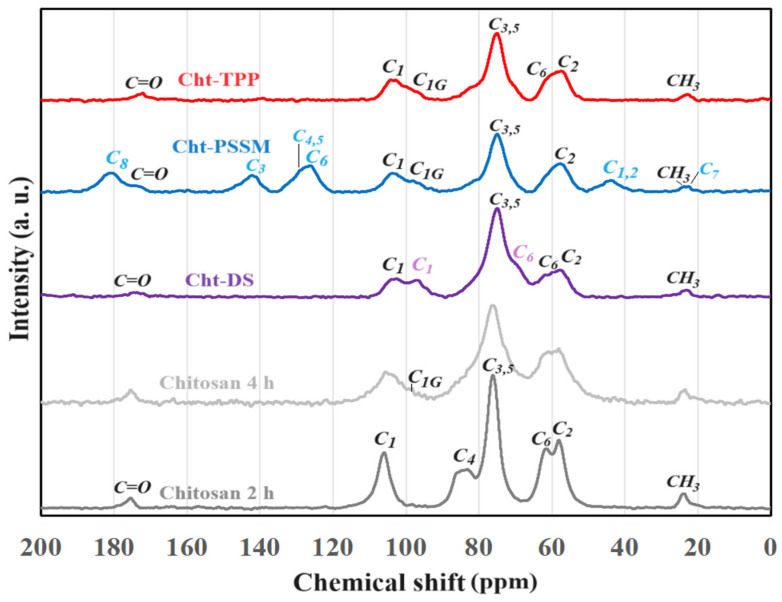
^13^C NMR spectra for milled chitosan and MCCL products. Peak labels refer to the carbon number, as assigned in the figures of Appendix A; *C_1G_* indicates the *C_1_* of glucosamine (with free OH and no glycosidic bridging).

Figure 4 presents the ^13^C NMR spectra of the examined samples, with peaks identified by the corresponding carbon numbers as delineated in Appendix A. The high-energy milled specimens, lacking a cross-linker addition, demonstrated decreased intensity and broadening of peaks with prolonged milling time. These alterations were ascribed to lattice disordering in chitosan, aligning with prior research [1]. Moreover, amorphization resulted in a slight reduction in chemical shift values of peaks by several ppm, observed in chitosan after extended high-energy milling (4 h) compared to samples milled for 2 h. Notably, a signal at 96 ppm (labeled *C_1G_*) corresponding to glucosamine’s *C_1_* with a free OH group (hydrolyzed glycosidic link) was identified in the sample milled for 4 h. This suggests that the intense mechanochemical treatment under the specified conditions reduced partially the length of chitosan chains [20].

The analysis of the Cht-DS pattern, including signals attributed to *C_1_* and *C_6_* of dextran’s pyranosic rings [41], indicates a high degree of amorphization in the cross-linked chitosan. Specifically, Cht-DS was prepared through 2 h of milling, resulting in a spectrum with broadened peaks and reduced intensity akin to chitosan milled for 4 h. This phenomenon may be attributed to the polyanionic nature of DS, facilitating interaction with chitosan via ionic bonding and consequently disrupting its crystal structure. Similarly, significant amorphization effects are evident in both Cht-PSSM and Cht-TPP. Furthermore, in the spectrum of Cht-PSSM, apart from peaks associated with the sulfonated polymer [42], a discernible signal related to glucosamine *C_1_* appeared at a chemical shift of 96 ppm. The same peak (at 95 ppm) was detected in the spectrum associated with Cht-TPP, while it is inferable that *C_1G_* is also present in the Cht-DS spectrum, albeit overlapped by the DS’ *C_1_* signal. Overall, NMR results confirm a decrease in chitosan chain length as one of the primary factors contributing to its substantially amorphous nature, thereby significantly enhancing the adsorption properties of the biopolymer.

### 3.3. Resistance in an Acidic Environment, Reusability, and Hardness of Mechanochemically Cross-Linked Chitosan

Previous experiments have confirmed that cross-linking has a beneficial effect on both the kinetics of RR2 adsorption and the adsorption capacity compared to pristine chitosan [6]. It has been observed that, at low pH, chitosan loses approximately 90% of its mass without cross-linking (using 1,2,7,8-diepoxyoctane), a loss which can only be reduced to zero with high fractions of the linker in chitosan. Consequently, the cross-linked adsorbent at pH 4 exhibited higher RR2 adsorption capacity and faster adsorption kinetics. Specifically, the low pH-induced protonation of most NH_2_ groups facilitated the dye’s binding through ionic interaction and hydrogen bonding. Additionally, cross-linking prevented chitosan from hydrolyzing in such acidic solutions, enabling effective adsorption of a significant amount of RR2 compared to pristine chitosan, which suffered from substantial dissolution in water, leading to poor performance. Building upon these findings, the subsequent part of this study was dedicated to confirming the acid resistance of MCCL chitosan and investigating the acid resistance and hardness of cross-linked chitosan tablets. These data are crucial for predicting whether the MCCL adsorbent can be utilized to prepare adsorption beds for continuous operations aimed at treating highly acidic industrial wastewater.

**Figure 5:**
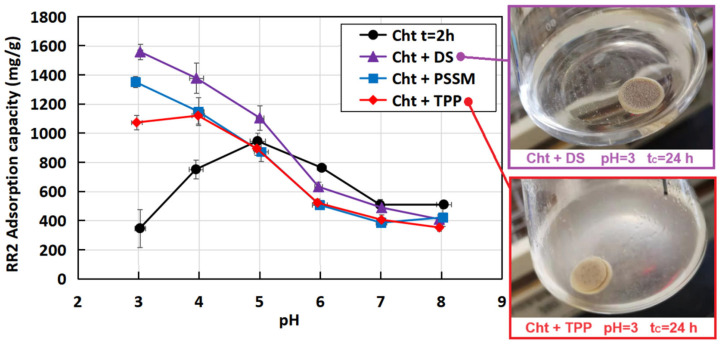
RR2 adsorption capacity of Cht-DS, Cht-PSSM, and Cht-TPP. The two photographs show the acidic aqueous solutions (pH = 3) in which cross-linked chitosan tablets were agitated for t_C_ = 24 h.

The graph in Figure 5 illustrates the RR2 adsorption capacity of powdery MCCL chitosan products as it varies with pH. The three cross-linked samples exhibit a decreasing trend in adsorption capacity with increasing pH, attributed to the deprotonation of chitosan’s amino groups, leading to a reduction in RR2 adsorption capacity. While the samples cross-linked with DS and PSSM reach their maxima at pH 3 (1559 and 1352 mg·g^−1^, respectively), it is noteworthy that the sample cross-linked with TPP reaches its maximum adsorption capacity at pH 4 (1121 mg·g^−1^), albeit only marginally higher than that at pH 3 (1074 mg·g^−1^). This difference is attributable to the limited protonation of TPP at pH 3, which destabilizes the cross-linked structure, causing partial hydrolysis and dissolution of the polysaccharide. The stability of MCCL chitosan is evident when comparing the three samples to the biopolymer milled for 2 h, which possesses similar particle dimensions and amorphization degree [19]. In the most acidic range (pH 3–4), milled chitosan exhibits low adsorption capacity due to significant mass loss in water, resulting from the hydrolysis of glycosidic bonds [6]. Its maximum RR2 adsorption capacity reaches 947 mg·g^−1^ at pH 5, declining at higher pH levels due to the deprotonation of amine groups.

The stability assessment of MCCL chitosan tablets in a pH 3 solution following 24 h of agitation (Figure 5) visually corroborates the previously discussed findings regarding cross-linked chitosan powder. The tablet fabricated with Cht-DS left the solution transparent, signifying minimal mass loss from the tablet; a comparable outcome was noted for the sample containing PSSM (photograph not provided). Conversely, Cht-TPP yielded a notably opaque solution, indicative of partial chitosan hydrolysis, which also resulted in decreased RR2 adsorption capacity in powder experiments. The tablet composed of chitosan was milled for 2 h and completely dissolved in a turbid solution after 24 h of agitation (photograph not provided).

**Figure 6:**
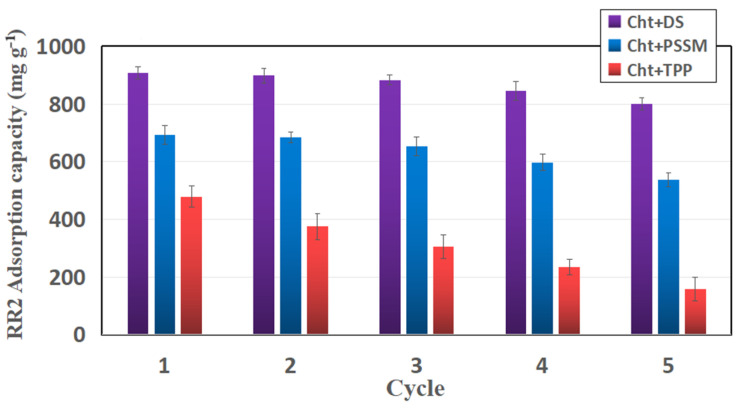
RR2 adsorption capacity of Cht-DS, Cht-PSSM, and Cht-TPP for each adsorption-regeneration cycle.

These findings were further substantiated by reusability tests conducted with tablets of the three adsorbents (Figure 6). It is noteworthy that the adsorption capacities in the first cycle are diminished compared to those measured in the powder form experiments (e.g., see values in Figure 5 at pH 3). One plausible explanation is that the tablet’s core remains inaccessible to the solution, thus not contributing to RR2 removal. Another rationale is the partial disruption of the porous structure of MCCL chitosan, resulting from the pressure applied during tablet preparation. Regarding Cht-TPP, the reduced adsorption performance could be attributed to the considerable loss of chitosan through hydrolysis (Figure 5). Chitosan hydrolyzation appears to be the most plausible explanation for the observed declining trend in RR2 adsorption capacity with increasing cycles. This decline is more pronounced for Cht-TTP and less for Cht-DS, affirming the superior performance of the latter adsorbent. It is important to note that another conceivable reason for the reduction in adsorption capacity in chitosan-based adsorbents is the more stable interaction between the adsorbate and the adsorption sites, hindering complete material regeneration [36].

Tablets fabricated with pristine chitosan, chitosan milled for 2 h, and the three chitosan products derived from the MCCL process underwent Vickers microhardness testing (Table 3). The findings demonstrated an escalation in hardness for the milled chitosan compared to its unmilled counterpart. This phenomenon can be attributed to the reduced particle size, thereby augmenting the inter-particle contact area within the compressed material, consequently enhancing its mechanical properties. Notably, the interaction with the cross-linkers substantially amplified the microhardness of the polysaccharide, with the attained values aligning consistently with those documented in a precedent study [43]. This substantiates that the formation of cross-links between positively charged amine groups and negatively charged acid groups of the cross-linkers (SO_3_^−^ in DS and PSSM, and PO_4_H_(3−n)_^n−^ in TPP) confers superior strength to the material. Significantly, the microhardness exhibited a consistent hierarchy with the RR2 adsorption capacity: DS > PSSM > TPP. This underscores the reliability of adsorption assessments conducted under low pH conditions in assessing the advancement of the MCCL reaction. In summary, the MCCL methodology holds promise as a cost-effective and readily scalable approach for producing efficient adsorbent beds with enhanced mechanical robustness for applications in water and wastewater treatment.

### 3.4. Application of Mechanochemically Cross-Linked Chitosan to the Regeneration of Rinsing Wastewater in the Chromium Electroplating Industry

The results of the RR2 adsorption experiments have clearly highlighted the outstanding performance of Cht-DS as an adsorbent, mainly due to its superior adsorption capacity, resistance in acidic solutions, reusability, and ability to form pellets with increased hardness when compared to pristine chitosan and other MCCL products. As a result, Cht-DS was selected for adsorption testing to regenerate simulated rinsing wastewater in the chromium electroplating industry. However, it is worth noting that DS could be a costly reagent on a large scale, as it is primarily used in medical applications. Therefore, Cht-PSSM could represent a more affordable alternative, as its properties do not differ significantly from those of Cht-DS. Moreover, environmental sustainability should be considered. For example, potential leaching of TPP could increase total phosphorus in the treated water, and this should be considered before selecting the cross-linker.

**Figure 7:**
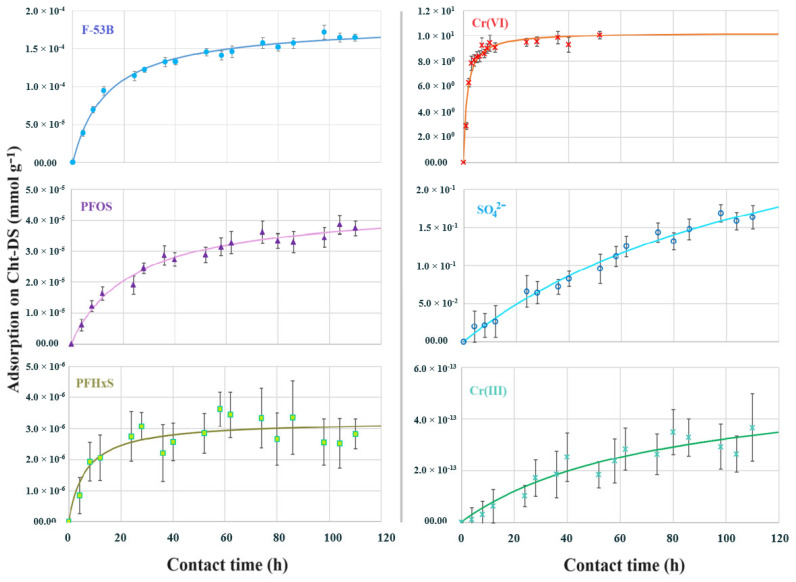
Kinetic evolution of adsorbent pollutants and components (F-53B, PFOS, PFHxS, Cr(VI), SO_4_^2−^, and Cr(III)) in simulated chromium electroplating rinsing wastewater. Straight lines represent the pseudo-second-order kinetic model. Experimental conditions: initial pH = 2.5, mass of the adsorbent Cht-DS = 50 mg, solution volume = 50 mL, initial solution composition: refer to Table 1.

Figure 7 illustrates the specific accumulation of each contaminant onto the Cht-DS surface as it varies with contact time. The results indicate that each component in the simulated wastewater is adsorbed at different rates. Specifically, Cr(VI) has the highest initial concentration (500 mg·L^−1^, 9616 μM) and is rapidly adsorbed by Cht-DS, reaching saturation in less than 20 h. The final concentration in the solution was found to be 120 μM, corresponding to a removal efficiency of 98.75%. In contrast, the three perfluorinated chemicals required much longer time to reach equilibrium, with adsorption occurring only after 100 h of contact time. F-53B (initial concentration 0.10 mg·L^−1^, 0.188 μM) was adsorbed by 93.93%, reaching a final concentration of 1.14 × 10^−2^ μM. The concentration of PFOS (initial value 0.02 mg·L^−1^, 0.04 mM) was reduced to 1.65 × 10^−3^ μM, achieving a removal of 95.87%. PFHxS (initial concentration 0.002 mg·L^−1^, 0.005 μM) reached a concentration of 6.30 × 10^−4^ M and a removal of 87.40%.

The sulfate adsorption rate was slower than the compounds mentioned earlier. Its initial concentration (400 mg·L^−1^, 4120 μM) decreased to 3949 μM, resulting in a removal of only 4.17%. Finally, as expected, the concentration of Cr(III) (initial value 15 mg·L^−1^, 288 μM) remained practically unchanged (286 μM), and therefore its removal percentage was almost negligible (0.86%).

Experimental data were fitted with the pseudo-first-order and the pseudo-second-order models (Table 4). Given the dispersed nature of the data, both models can roughly predict the observed kinetic trends. However, the generally higher values of R^2^ and lower values of χ^2^ for the pseudo-second-order model suggest that it may better fit the data. Moreover, this model aligns with previous kinetic studies on chitosan, which emphasized the chemisorptive nature of the interaction between the adsorbent surface and the adsorbate, primarily due to the formation of ionic and hydrogen bonds [44,45].

Pollutants are adsorbed via ionic/hydrogen bonding interactions with anionic contaminants, owing to the high protonation percentage of the amine groups [46]. Furthermore, PFAS can also interact with chitosan through hydrophobic interactions with the perfluorinated tail [47]. Thus, the anticipated low efficacy of Cht-DS in adsorbing Cr(III) stemmed from the repulsion of Cr(III) cations by the positive charge on the chitosan surface. Conversely, the limited adsorption of sulfates warrants further discussion. While prior research has demonstrated chitosan’s capacity to uptake sulfates from aqueous solutions [48], the observed low removal suggests a slow adsorption rate compared to the other investigated anionic adsorbates. Specifically, sulfate anions have tetrahedral symmetry, lacking a permanent dipolar moment, unlike dichromate and PFAS, potentially resulting in slower SO_4_^2−^ mobility at the boundary layer interface with the adsorbent.

Another noteworthy consideration is the adsorption behavior of the three perfluorinated chemicals. Typically, the most hydrophobic congeners, usually with longer perfluorinated chains, outcompete shorter ones over longer contact times, inducing detachment from the chitosan surface and occupying adsorption sites with stronger hydrophobic interactions [47]. However, this phenomenon was not observed in the present study, likely due to substantial differences in concentrations of the PFAS and, importantly, their low initial concentrations, as well as the presence of other inorganic components that did not trigger competition for the Cht-DS sites. This underscores the necessity for experimental confirmation of potential phenomena such as competition, especially when treating complex mixtures of pollutants.

Lastly, it is noteworthy that the contained costs of the proposed mechanochemical method for synthesizing cross-linked chitosan adsorbents, primarily due to its solvent-freeness, allow for the preparation of either expendable adsorbents (in small water treatment plants) or regenerable ones (in larger plants). Careful economic considerations should be made to assess the most technically and economically effective option. In conclusion, the experiment with simulated wastewater confirms the feasibility of using Cht-DS or other MCCL chitosan-based adsorbents to remove multiple toxic pollutants in a single adsorption step, enabling the regeneration of rinsing wastewater for further use in electroplating plants.

## 4. Conclusions

This study introduced a solid-state mechanochemical method for cross-linking chitosan with polyanionic compounds. Experimental results for the mechanochemical reaction conditions indicate that a short duration (2–4 h) of high-energy ball milling of the powder reagents, with low mass percentages of the cross-linker (<30%*w*/*w*), could effectively produce products with the desired properties. The optimal product was obtained with only 10%*w*/*w* dextran sulfate and milled for 2 h. Stability tests in pH 3 solution confirmed negligible mass loss for DS and PSSM cross-linked chitosan tablets, unlike TPP-cross-linked and milled chitosan, which dissolved. This material exhibited a remarkable adsorption capacity for the organic dye RR2 at low pH, indicating significant resistance to strongly acidic environments. The Cht-DS demonstrated the highest RR2 adsorption capacity, reaching 1559 mg·g^−1^ at pH 3. The Cht-PSSM exhibited a slightly lower RR2 adsorption capacity of 1352 mg·g^−1^, while the Cht-TPP product had the lowest adsorption capacity of 1074 mg·g^−1^.

Additionally, tablets prepared with mechanochemically cross-linked chitosan showed an almost threefold enhancement in microhardness compared to the pristine biopolymer. NMR analysis revealed increased amorphization and shortening of chitosan chain length in the cross-linked products. FTIR spectroscopy confirmed the stiffening of the chitosan structure due to the formation of ionic bonds between chitosan’s protonated amine groups and cross-linker acid groups. These results validate the feasibility of mechanochemical cross-linking as a green approach to enhance chitosan resistance in acidic environments and its mechanical properties. This was further supported by experiments with simulated rinsing wastewater from the chrome electroplating industry, contaminated by both PFAS and Cr species and characterized by an extremely low pH. Cht-DS demonstrated superior adsorption performance compared to pristine chitosan and other MCCL products. Cr(VI) was rapidly adsorbed, achieving a 98.75% removal in under 20 h. The PFAS, i.e., F-53B, PFOS, and PFHxS, required over 100 h for removals of 93.93%, 95.87%, and 87.40%, respectively. The study found that the mechanochemically cross-linked chitosan exhibited a significant increase in adsorption capacity for other pollutants, such as Cr(VI) and PFOS, with removal efficiencies of 98.75% and 95.87%, respectively. Such outcomes support the potential application of mechanochemically cross-linked chitosan as an adsorbent with improved properties for removing contaminants from industrial wastewater characterized by harsh acidity. Future investigations should be directed to tackle possible problems that may be encountered in the application of this technology. For instance, further studies should be aimed at confirming the scalability of the mechanochemical cross-linking reaction in pilot- and large-scale high-energy mills. Additional research should also focus on the mechanical properties of the mechanochemically cross-linked chitosan and possible methods to enhance them, aiming to prepare suitable beds for industrial adsorption columns.

## Figures and Tables

**Table 1:** Composition of the simulated chrome-plating wastewater.

Component	Value	Reference
Cr_2_O_7_^2−^	500.0 mg·L^−1^ (9616 mM)	[25]
Cr^3+^	15.00 mg·L^−1^ (288 mM)	[25]
PFOS	0.020 mg·L^−1^ (0.04 mM)	[26]
PFHxS	0.002 mg·L^−1^ (0.005 mM)	[26]
F-53B	0.100 mg·L^−1^ (0.188 mM)	[26]
SO_4_^2−^	400.0 mg·L^−1^ (4120 mM)	[25]
pH	2.5	[25]

**Table 2:** Specific surface area of pristine chitosan and milled samples.

Sample	Specific Surface Area (m^2^ g^−1^)
Chitosan	0.922
High-energy milled chitosan (2 h)	2.073
Chitosan + 10%*w*/*w* DS (2 h)	1.960
Chitosan + 30%*w*/*w* PSSM (2 h)	1.776
Chitosan + 30%*w*/*w* TPP (4 h)	1.339

**Table 3:** Vickers microhardness for the investigated specimens.

Sample	Microhardness (MPa)
Chitosan	266 ± 56
High-energy milled chitosan (2 h)	329 ± 38
Chitosan + 10%*w*/*w* DS (2 h)	690 ± 88
Chitosan + 30%*w*/*w* PSSM (2 h)	667 ± 90
Chitosan + 30%*w*/*w* TPP (4 h)	587 ± 71

**Table 4:** Parameters and statistical indices for the pseudo-first-order and the pseudo-second-order models estimated for the six components of the simulated wastewater.

Component	Pseudo-First-Order Model ^*a^				Pseudo-Second-Order Model ^*b^			
	q_e_ (mmol·g^−1^)	k (h^^−1^^)	R^2^	χ^2^	q_e_ (mmol·g^−1^)	ν0 (g·mmol^−1^ h^−1^)	R^2^	χ^2^
Cr(VI)	9.41	0.480	0.9746	0.1803	10.3	7.59	0.9605	0.2798
SO42−	0.234	0.0114	0.9847	4.68 × 10^−5^	0.373	0.0028	0.9846	4.70 × 10^−5^
Cr(III)	3.84 × 10^−13^	0.0182	0.9142	1.23 × 10^−27^	5.69 × 10^−13^	7.51 × 10^−15^	0.9112	1.27 × 10^−27^
F-53B	1.57 × 10^−4^	0.0596	0.9685	7.22 × 10^−11^	1.83 × 10^−4^	1.34 × 10^−5^	0.9901	1.83 × 10^−4^
PFOS	3.62 × 10^−5^	0.0386	0.9703	3.84 × 10^−12^	4.49 × 10^−5^	1.84 × 10^−6^	0.9793	2.68 × 10^−12^
PFHxS	2.95 × 10^−6^	0.107	0.8311	1.45 × 10^−13^	3.24 × 10^−6^	5.00 × 10^−7^	0.8143	1.60 × 10^−13^

*^a^ q_t_ = q_e_ (1 − e^kt^). *^b^ q_t_ = (q_e_·ν_0_·t)/(q_e_ + ν_0_·t).

**Figure A3:**
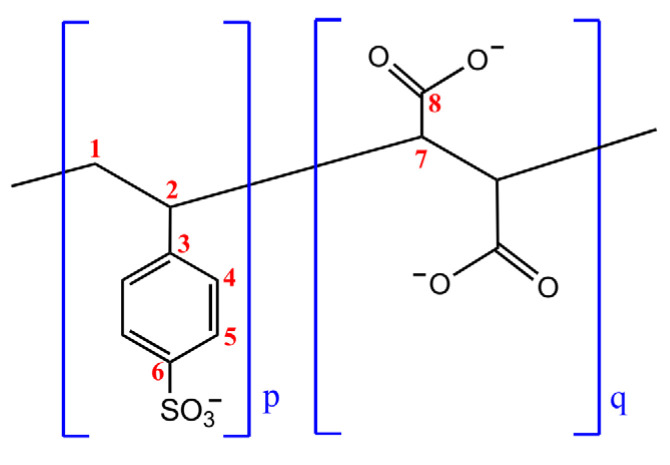
Poly[4-styrenesulfonic acid-co-maleic acid].

## Data Availability

The original contributions presented in the study are included in the article; further inquiries can be directed to the corresponding authors.

## Appendix A

Chemical structures of the main chemicals used in the present study.
